# Supplementary material for: Comparative Evaluation of the Foot-and-Mouth Disease Virus Permissive LF-BK αVβ6 Cell Line for Senecavirus A Research
Source: Viruses. 2022 Aug 25;14(9):1875. doi: 10.3390/v14091875 (PMC9503874; doi:10.3390/v14091875)
Supplement: Supplementary file 1 [file viruses-14-01875-s001.zip › Supplemental Table S1.pdf]

Table S1: SVA and FMDV geometric mean neutralizing antibody titers (log_{10}) collected on days 0 and 35 for FMD vaccinated and unvaccinated swine.

Animal ID	SVA neutralizing antibody titers (log_{10})		FMDV neutralizing antibody titers (log_{10})		FMD vaccinated
	Day 0	Day 35	Day 0	Day 35	
51822	0.6	1.2	0.6	1.2	Yes
51823	0.6	1.5	0.6	1.8	Yes
51824	0.6	1.2	0.6	2.4	Yes
51825	1.2	1.2	0.6	1.8	Yes
51826	1.2	0.9	0.6	2.1	Yes
51827	1.8	0.9	0.6	3.0	Yes
51828	0.9	1.5	0.6	1.8	Yes
51829	1.8	1.8	0.6	0.9	Yes
51830	1.2	0.6	0.6	1.8	Yes
51831	0.9	1.2	0.6	2.1	Yes
51832	0.9	1.2	0.6	1.2	Yes
51833	0.9	0.9	0.6	1.2	Yes
51834	0.6	0.6	0.6	2.4	Yes
51835	0.6	1.2	0.6	2.1	Yes
51836	1.2	1.2	0.6	2.1	Yes
51837	0.6	1.2	0.6	2.1	Yes
51838	0.9	0.9	0.6	2.4	Yes
51839	0.9	0.6	0.6	0.9	Yes
51840	0.9	1.2	0.6	1.5	Yes
51841	1.2	0.6	0.6	1.2	Yes
51842	0.6	0.6	0.6	1.8	Yes
51843	1.2	1.2	0.6	1.8	Yes
51844	0.9	0.9	0.6	1.5	Yes
51845	2.4	0.9	0.6	1.5	Yes
51846	0.9	1.2	0.6	0.6	No
51847	1.2	0.6	0.6	1.5	Yes
51848	1.2	1.2	0.6	1.8	Yes
51849	0.9	1.5	0.6	0.9	Yes
51850	0.6	0.9	0.6	0.6	No
51851	0.6	0.6	0.6	1.2	Yes
51852	1.2	0.6	0.6	0.6	No
51853	0.6	0.9	0.6	1.8	Yes

51854	0.6	0.9	0.6	1.8	Yes
51855	0.9	1.2	0.6	0.6	No
51856	0.6	0.6	0.6	0.6	No
51857	0.6	0.9	0.6	0.6	No
51858	1.2	0.6	0.6	0.6	No
51859	0.6	0.6	0.6	0.6	No
51860	0.6	0.6	0.6	0.6	No
51861	0.9	1.2	0.6	0.6	No
53546	0.9	2.1	0.6	2.7	Yes
53547	0.6	0.6	0.6	1.8	Yes
53548	0.9	N/A	0.6	N/A	No
53549	0.6	0.6	0.6	2.4	Yes
53550	1.2	N/A	0.6	N/A	No
53551	0.9	1.8	0.6	2.4	Yes
53552	0.6	0.6	0.6	2.4	Yes
53553	0.6	0.6	0.6	2.7	Yes
53554	0.6	1.2	0.6	2.1	Yes
53555	0.6	0.9	0.6	1.8	Yes
53556	1.2	N/A	0.6	N/A	No
53557	0.9	0.9	0.6	1.5	Yes
53558	0.6	0.9	0.6	2.4	Yes
53559	1.2	1.5	0.6	2.4	Yes
53560	1.2	0.6	0.6	2.7	Yes
53561	0.6	0.6	0.6	0.6	Yes
53562	1.2	1.2	0.6	1.5	Yes
53563	0.9	0.9	0.6	2.1	Yes
53564	1.2	1.8	0.6	2.4	Yes
53565	0.6	0.9	0.6	0.6	No
53566	0.6	0.6	0.6	2.4	Yes
53567	1.2	0.6	0.6	2.7	Yes
53568	1.2	1.2	0.6	1.5	Yes
53569	1.2	0.6	0.6	0.6	No
53570	1.2	1.2	0.6	0.9	Yes
53571	0.9	0.6	0.6	2.1	Yes
53572	0.6	0.9	0.6	1.2	Yes
53573	1.2	0.9	0.6	0.6	Yes
53574	0.6	1.2	0.6	3.0	Yes
53575	1.2	1.2	0.6	0.6	No
53576	1.2	1.2	0.6	0.6	No
53577	0.6	N/A	0.6	N/A	No
53578	0.6	1.2	0.6	2.1	Yes
53579	1.2	0.6	0.6	1.2	Yes
53580	0.9	0.6	0.6	2.7	Yes

53581	0.9	0.6	0.6	N/A	Yes
53582	0.6	1.2	0.6	2.7	Yes
53583	0.6	1.2	0.6	2.4	Yes
53584	1.2	1.2	0.6	2.4	Yes
53585	1.2	N/A	0.6	N/A	No
53586	1.2	1.2	0.6	2.1	Yes
53587	0.6	1.2	0.6	3.0	Yes
53588	1.2	0.9	0.6	1.5	Yes
53589	0.9	0.9	0.6	0.6	No
53590	1.2	1.2	0.6	3.0	Yes
